# Comparative Mitogenome Analysis of the Genus *Trifolium* Reveals Independent Gene Fission of *ccmFn* and Intracellular Gene Transfers in Fabaceae

**DOI:** 10.3390/ijms21061959

**Published:** 2020-03-13

**Authors:** In-Su Choi, Tracey A. Ruhlman, Robert K. Jansen

**Affiliations:** 1 Department of Integrative Biology, University of Texas at Austin, Austin, TX 78712, USA; truhlman@austin.utexas.edu (T.A.R.); jansen@austin.utexas.edu (R.K.J.); 2 Centre of Excellence in Bionanoscience Research, Department of Biological Sciences, Faculty of Science, King Abdulaziz University, Jeddah 21589, Saudi Arabia

**Keywords:** legumes, clover, organelle genetics, mitochondria, endosymbiotic gene transfer, gene fission

## Abstract

The genus *Trifolium* is the largest of the tribe Trifolieae in the subfamily Papilionoideae (Fabaceae). The paucity of mitochondrial genome (mitogenome) sequences has hindered comparative analyses among the three genomic compartments of the plant cell (nucleus, mitochondrion and plastid). We assembled four mitogenomes from the two subgenera (*Chronosemium* and *Trifolium*) of the genus. The four *Trifolium* mitogenomes were compact (294,911–348,724 bp in length) and contained limited repetitive (6.6–8.6%) DNA. Comparison of organelle repeat content highlighted the distinct evolutionary trajectory of plastid genomes in a subset of *Trifolium* species. Intracellular gene transfer (IGT) was analyzed among the three genomic compartments revealing functional transfer of mitochondrial *rps1* to nuclear genome along with other IGT events. Phylogenetic analysis based on mitochondrial and nuclear *rps1* sequences revealed that the functional transfer in Trifolieae was independent from the event that occurred in robinioid clade that includes genus *Lotus*. A novel, independent fission event of *ccmFn* in *Trifolium* was identified, caused by a 59 bp deletion. Fissions of this gene reported previously in land plants were reassessed and compared with *Trifolium*.

## 1. Introduction

Plant cells comprise three genomic compartments (i.e., nucleus, mitochondrion and plastid). Unlike the typically conservative plastid genome (plastome) [1,2], plant mitochondrial genomes (mitogenome) display drastic evolutionary plasticity in size, content and structure, intracellular gene transfer (IGT) and interspecific horizontal gene transfer [3,4,5,6,7,8]. Substitution rates of mitochondrial protein coding genes, however, are the most conservative among the three genomic compartments [9]. In angiosperms, the relative rate of synonymous substitutions of mitogenome, plastome and nuclear genome is 1:3:16 [10].

Extensive gene loss and IGT of organelle DNA to the nucleus occurred in the early stages of endosymbiosis [11]. Nuclear genome sequences that originate from the mitogenome and plastome are referred to as nuclear mitochondrial DNA sequences (NUMTs) and nuclear plastid DNA sequences (NUPTs), respectively [12,13]. Transfer of mitochondrial DNA to the nuclear genome is an ongoing process in both of plants and animals but functional transfer of mitochondrial genes has almost ceased in animals [14]. Functional transfer of mitochondrial genes in plants has often involved ribosomal protein or succinate dehydrogenase genes [5]. Transfer of mitochondrial genes to the nuclear genome cannot substitute function of the original mitochondrial copy unless the nuclear copy acquires the appropriate expression and targeting signals [15]. Before the acquisition of regulatory signals, NUMTs must survive mutational decay in nuclear genome, which limits the lifespan of the nonfunctional sequences [16]. Mitochondrial IGT events may be successful or unsuccessful in terms of functionality and the phylogenetic distribution of pseudogenization and deletion of mitochondrial genes can be assessed in descendant lineages [17].

Following functional transfer, NUMTs attain higher substitution rates than their mitochondrial counterparts [14] because of substantial differences in the synonymous substitution rate between mitochondrial and nuclear genomes in plants [10]. Hence, functional transfer of mitochondrial genes into the nucleus is often detected by the presence of intact but highly diverged copies in nuclear genome compared to mitochondrial copies [18,19]. On rare occasions, functional transfers of mitochondrial genes exhibit an intriguing situation in which the nuclear and mitochondrial genomes contain different portions of the coding region resulting from mitochondrial gene fission and IGT (e.g., *rpl2* in many of eudicots) [20]. Szafranski [21] named this process “intercompartmental piecewise gene transfer.” In plant mitogenome evolution, the protein that most commonly undergoes gene fission is cytochrome c maturation protein *ccmF* [22,23,24,25].

In *Escherichia coli*, the eight *ccm* genes (*ccmA*-*H*) are clustered in a single locus [26]. In most plants, three *ccm* genes (*ccmA*, *ccmE* and *ccmH*) have been transferred from the mitogenome to the nuclear genome, two (*ccmD* and *ccmG*) were lost and four (*ccmB*, *ccmC*, *ccmFc* and *ccmFn*) remain in the mitochondrion [27]. Since the fission of *ccmF* into *ccmFc* and *ccmFn* is shared by liverworts and seed plants [3], this event happened early in land plant evolution. In addition, there were independent fissions of *ccmF* in several lineages of land plants, including fission of *ccmFc* into *ccmFc1* and *ccmFc2* in *Marchantia* [22] and fissions of *ccmFn* into *ccmFn1* and *ccmFn2* in Brassicaceae [23,24] and *Allium* (Amaryllidaceae) [25].

Fabaceae are the third largest angiosperm family with approximately 20,000 species in six subfamilies [28]. Most species diversity occurs in subfamily Papilionoideae, which includes many economically important species [29]. The inverted repeat (IR) lacking clade (IRLC) is one of the major groups of Papilionoideae, which is defined by absence of the canonical plastome IR (~25 kb) [30]. Plastome studies of the IRLC elucidated several rare evolutionary phenomena, including high degree of genome rearrangement [31], localized hypermutation [32], genome size expansion with accumulation of dispersed repeats and unique sequences of unknown origin [33,34,35] and re-acquisition of a large IR [36]. However, mitogenome evolution in IRLC is poorly understood and represented by only two species, *Vicia faba* (tribe Fabeae) [37] and *Medicago truncatula* (tribe Trifolieae) [38]. In Trifolieae, a study of the mitochondrial *rps1* gene documented the existence of functional nuclear copies and putatively pseudogenized mitochondrial copies from three genera (*Medicago*, *Melilotus* and *Trigonella*) [39]. Deletion of mitochondrial *rps1* was also identified from another papilionoid species, *Lotus japonicus* [40]. The status of mitochondrial *rps1* across Trifolieae and related taxa has not been examined until recently. Parallel losses of several mitochondrial genes in Fabaceae were revealed in a previous study, however, whether the losses represent a single ancestral IGT or multiple IGTs was not determined [8].

*Trifolium* is the largest genus (ca. 250 species) of the tribe Trifolieae [41] and is divided into two subgenera (*Chronosemium* and *Trifolium*) [42]. Trifolieae belong to the IRLC and are closely related to Fabeae [30]. Several evolutionary studies of Trifolieae plastid [31,33,34] and nuclear [43,44,45,46] genomes have been conducted but mitogenome comparisons of *Trifolium* have been neglected. In this study, gene content, size and repeat structure of mitogenomes of four *Trifolium* species from the two subgenera *Chronosemium* (*T. aureum* and *T. grandiflorum*) and *Trifolium* (*T. meduseum* and *T. pratense*) were examined and compared to related papilionoid species.

## 2. Results

### 2.1. Mitogenome Features of Four Trifolium Species

**Figure 1:**
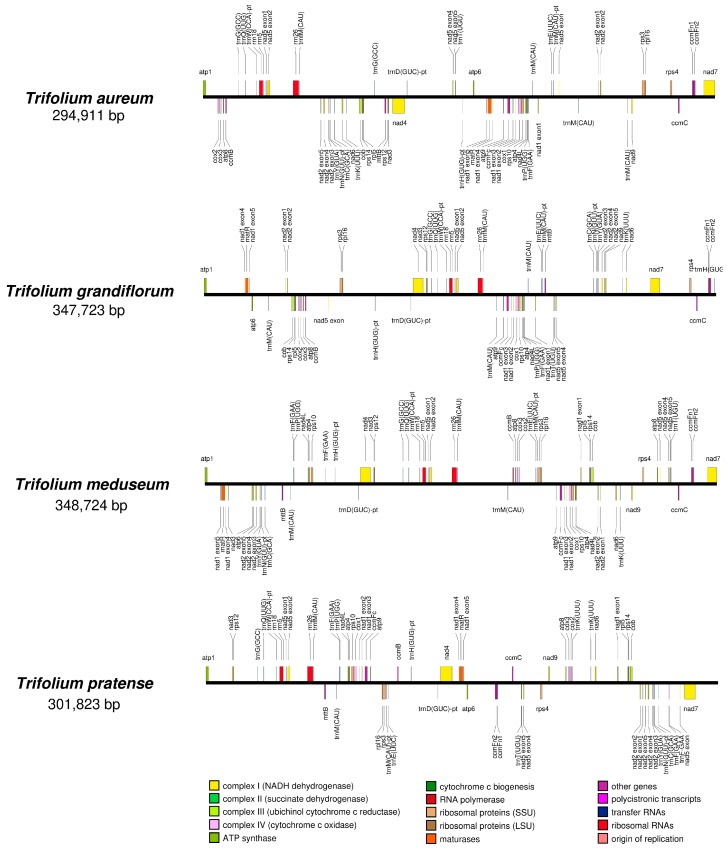
Linear mitogenome maps of four *Trifolium* species. Fragmented genes caused by duplication or pseudogenization are not depicted. pt indicates tRNAs of plastid origin.

For each of four *Trifolium*, a single chromosome was assembled that contained all expected mitochondrial coding sequences. The length of the four mitogenomes varied from to 294,911 to 348,724 bp (Table 1). The GC content was conserved among the species at 44.9–45.2 %. Gene content was identical with three rRNAs, 16 tRNAs and 32 protein coding genes while gene order was distinct for each species (Figure 1).

**Figure 2:**
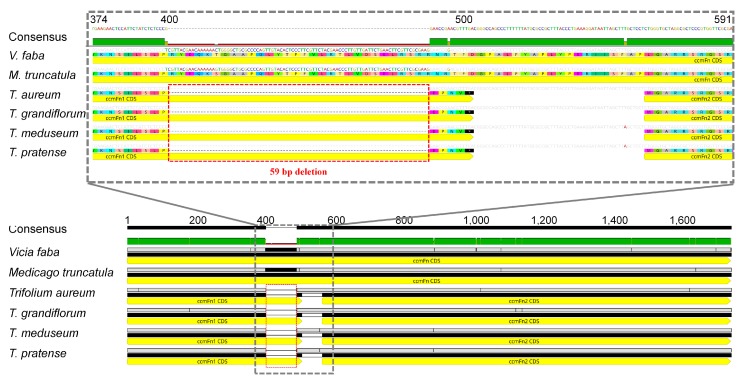
Fission of *ccmFn* in four *Trifolium*. Alignment of six *ccmFn* sequences of mitogenomes from species in inverted repeat lacking clade. The region (grey dashed rectangle, aligned positions 374-591) showing the 59 bp deletion (red dotted box) is enlarged above. Translated amino acid alignments are presented below corresponding nucleotide sequence alignments. Nucleotide coordinates are indicated above consensus of alignment. Sequence identity is shown below consensus (green = 100%, yellow-green = at least 30% and under 100%, red = below 30%).

Gene and intron content comparison with other published mitogenomes revealed one gene loss (*rps1*) (Figure S1), which was shared with *Lotus* and two cis-spliced intron losses (ccmFci829 and rps3i174) that were exclusive to *Trifolium* (Figure S2). Sequence alignment of *ccmFn* from *Trifolium* with other IRLC genera revealed a 59 bp deletion that resulted in a frame shift and premature stop codon (Figure 2). A putative downstream start codon for a second open reading frame (ORF) (*ccmFn2*) was also identified.

### 2.2. Repeat Composition of Organelle Genomes in Trifolium

**Figure 3:**
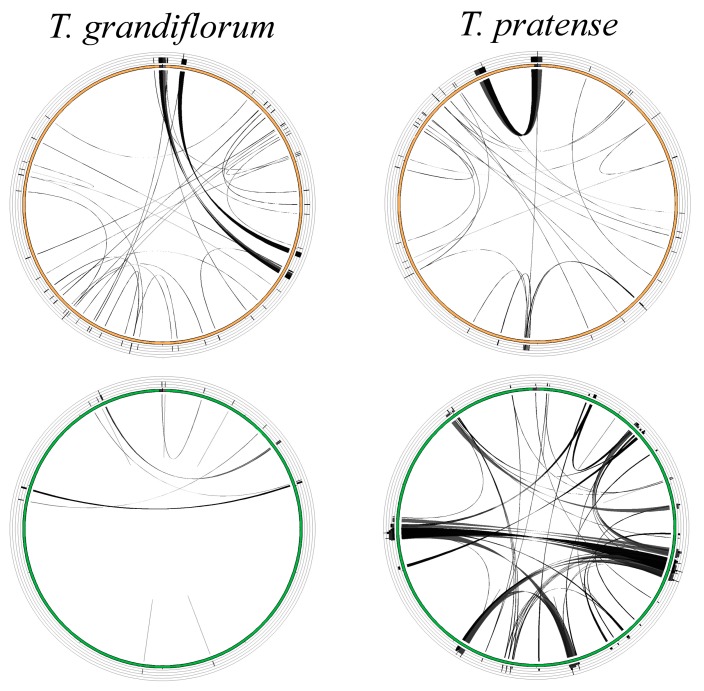
Distribution pattern of dispersed repeat sequences in circular representations of organelle genomes of two *Trifolium* species. Each ribbon represents a BLAST hit for a pair of dispersed repeats. Brown circles are mitogenomes and green circles are plastomes. Multiple hits in a single region are indicated by histograms in outer concentric rings. Data for repetitive sequences of all published organelle genomes of *Trifolium* is available in Table 2.

Repeat sequences were estimated four mitogenomes and thirteen plastomes (Table 2). The amount of repetitive sequences in mitogenomes was not highly variable (6.6~8.6 %). In contrast, the amount of repetitive DNA in plastomes was highly variable (4.4%~20.7%) and can be divided into two non-overlapping ranges that corresponded to two groups of two sections (subgen. *Chronosemium* sect. *Chronosemium* and subg. *Trifolium* sect. *Paramesus*, 4.4%~5.2 %) and five sects. of subg. *Trifolium* (*Lupinaster*, *Trichocephalum*, *Trifolium*, *Vesicastrum* and *Trifoliastrum*, 10.7%~20.7 %). The contrasting repeat composition between organelle genomes was particularly evident in *T. pratense*, which had smallest amount of repeat sequence in its mitogenome and the largest amount in its plastome (Figure 3; Table 2).

### 2.3. Intracellular Gene Transfer (IGT) in Trifolium

**Figure 4:**
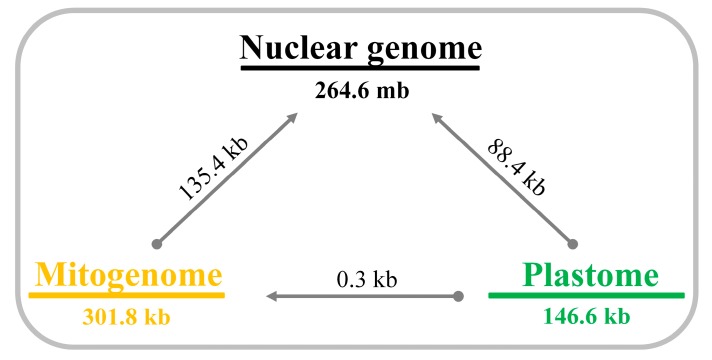
Shared DNA among three different genomic compartments in *Trifolium pratense*. Size of each genome is presented below the bar. Values on arrows represent the amount of shared DNA. Round end of arrow represents query sequence and pointed end represents subject sequence in BLAST analyses.

The extent of IGT among the three genomic compartments was analyzed in *T. pratense* by BLAST (Figure 4; Table 3). The amount of DNA shared between the two organelle genomes was very low (0.3 kb). The organelle genomes shared considerable DNA with the nuclear genome and GC content of shared DNA reflected the compartment of origin (45.8% for mitogenome and 35.1% for plastome). In general, BLAST hits between nuclear and organelle genomes were very short and had high sequence identity (Table 3).

A long contiguous region (348.5 kb) was identified from chromosome 4 of *T. repens* (position: 72,476,623–72,825,180) that shared substantial DNA with the mitogenome of *T. meduseum* (Figure S3). This sequence had a high GC content (44.3%) compared to the entire chromosome 4 (33.2%).

### 2.4. Multiple Functional Transfers of Mitochondrial rps1 in Papilionoideae

**Figure 5:**
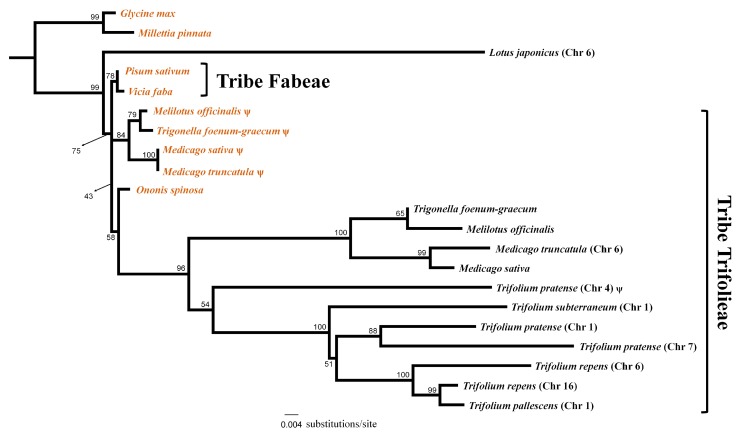
Maximum likelihood phylogeny of *rps1* gene. Bootstrap values are indicated at nodes. Sequences from mitogenome are indicated in brown. Sequences from nuclear genome are indicated as black and chromosome (Chr) numbers are specified in the parentheses when available.

A phylogenetic analysis of nuclear and mitochondrial copies of *rps1* for papilionoid legumes was conducted (Figure 5). Nuclear genomes of two *Trifolium* species (*T. pratense* and *T. repens*) (Table S1) included multiple *rps1* copies. Nuclear copies of *rps1* were placed in two separate positions, one that included *Lotus* sister to the taxa in the tribes Fabeae and Trifolieae and the second with four genera of the tribe Trifolieae (*Trigonella*, *Melilotus*, *Medicago* and *Trifolium*). Branch lengths for the nuclear copies of *rps1* were substantially longer than mitochondrial copies indicating accelerated substitution rates. The Trifolieae was monophyletic but the branch leading to the tribe was very short and the bootstrap value (BS = 43%) was low. In Trifolieae, the mitochondrial *rps1* sequences formed a paraphyletic grade sister to a monophyletic group of nuclear *rps1* (BS = 96%).

### 2.5. Fission of ccmF in Land Plants

To investigate the phylogenetic distribution of the fission of *ccmFn* and conservation of two ORFs *ccmFn1* and *ccmFn2* in *Trifolium*, mitochondrial *ccmF* sequences were assembled using available next-generation sequencing (NGS) reads (Table S2). The expanded taxon sampling confirmed the adjacency of the ORFs *ccmFn1* and *ccmFn2* and that the fission was restricted to *Trifolium*. All examined *Trifolium* species shared the *ccmFc* intron loss. Draft nuclear genome sequences of four species of *Trifolium* (*T. subterraneum*, *T. pratense*, *T. pallescens* and *T. repens*) were examined for intact copies of *ccmFn1* and *ccmFn2*. Fragments of sequences similar to *ccmFn1* and *ccmFn2* were identified in *T. subterraneum* and *T. pratense* but no intact copies were detected. However, intact copies both of *ccmFn1* and *ccmFn2* from *T. pallescens* (chromosome 4) and *T. repens* (chromosomes 4 and 9) were identified and were adjacent as in mitogenomes of *Trifolium*. Eleven *ccmFn* sequences (eight mitochondrial and three nuclear copies) were detected in *Trifolium* (Figure S4a). All nuclear copies were identical to their corresponding mitochondrial copy. Among mitochondrial copies, only three *Trifolium* species (*T. aureum*, *T. grandiflorum* and *T. pallescens*) showed unique sequence and the remaining sequences in the other five species were identical to each other in the coding region (Figure S4b).

Fission of *ccmFc* was analyzed in three species of *Marchantia* and two other genera of the Marchantiales. Sequence alignment revealed that a single nucleotide deletion caused *ccmFc* fission in one species of *Marchantia*, *M. paleacea* (Figure S5).

Examination of *ccmFn* fission in Brassicaceae included 17 taxa (Table S2). The *ccmF* genes were assembled from Cleomaceae (*Cleome violacea*), the sister family of Brassicaceae and two early diverging Brassicaceae genera (*Aethionema* and *Odontarrhena*). The fission of *ccmFn* was shared by all Brassicaceae except *Aethionema* and in all cases *ccmFn1* and *ccmFn2* were found in different loci. *Odontarrhena argentea* was the only member of the Brassicaceae that lost the *ccmFc* intron.

**Figure 6:**
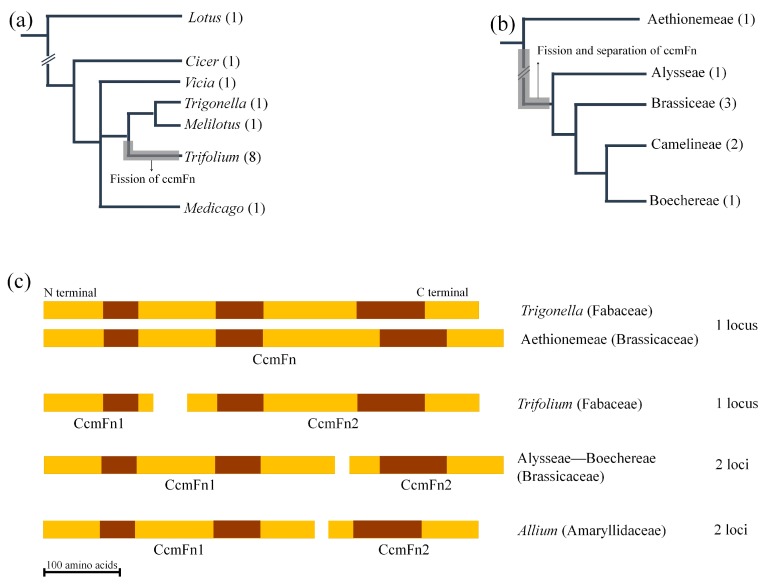
Fission events of *ccmFn* in angiosperms. (**a**) Examined genera of Fabaceae are plotted on cladogram from Ellison et al. [42]. Number of examined species is indicated in the parenthesis. The branch where the fission occurred is marked with grey bar. (**b**) Examined tribes of Brassicaceae are plotted on cladogram from Huang et al. [47]. Number of examined genera is indicated in the parenthesis. The branch where the fission and separation occurred is marked with grey bar. (**c**) *CcmFn* gene organization compared among various taxa of angiosperms. Dark brown box indicates conserved domains.

The phylogenetic position of *ccmFn* fission and separation in Fabaceae and Brassicaceae (Table S2), were plotted on cladograms of each of family (Figure 6a,b). The location of the breakpoint of *ccmFn* fission was also compared among the three families Fabaceae, Brassicaceae and Amaryllidaceae (Figure 6c). The fission occurred in different locations in the gene within each family and occurred in a more basal position in Brassicaceae than Fabaceae. The separation of *ccmFn1* and *ccmFn2* only occurred in Amaryllidaceae and Brassicaceae.

## 3. Discussion

### 3.1. Contrasting Evolutionary Trajectories of Trifolium Organelle Genomes

*Trifolium* mitogenomes (294,911 to 348,724 bp) (Table 1) are similar in size to the other Trifolieae genus *Medicago* (271,618 bp), which has the smallest currently sequenced papilionoid mitogenome [8]. Mitogenomes of *Trifolium* have relatively little repetitive DNA (6.6–8.6%) (Table 2) compared to mitogenomes of other Papilionoideae species (2.9–60.6%) [8]. This low repeat content in the mitogenome is in contrast to the plastome of some *Trifolium* species. The acquisition of numerous, novel repeat sequences and drastic rearrangement in the plastome of *T. subterraneum* and related species has been reported [31,33,35]. Increased taxon sampling by Sveinsson and Cronk [34] revealed that plastome expansion is shared by five sections, referred to as the “refractory clade” in subgenus *Trifolium* (*Lupinaster*, *Trichocephalum*, *Trifolium*, *Vesicastrum* and *Trifoliastrum*). The distinct evolutionary trajectory of organelle genomes in the genus is particularly evident in *T. pratense*, which has the lowest percentage of repetitive DNA in the mitogenome and the highest in the plastome as well as the most highly rearranged structure (Table 2 and Figure 3). In plant mitogenomes, accumulation of repeats, genome expansions and rearrangements may be a consequence of error-prone DNA repair mechanisms such as nonhomologous end-joining or break-induced-replication [48,49,50]. In Geraniaceae, a correlation between nonsynonymous substitution rates for DNA replication, recombination and repair (DNA-RRR) genes and plastome complexity was reported [51]. The plastome-specific increase in repeat complexity in the *Trifolium* refractory clade may be the result of disruption of ‘plastid specific’ DNA-RRR-protein genes, some of which are targeted to both mitochondria and plastids [7]. More comprehensive taxon sampling that includes data from all three plant genomic compartments of *Trifolium* is required to test this hypothesis.

### 3.2. Multiple Functional Transfers of the Mitochondrial rps1 Gene to the Nucleus in Papilionoideae

An earlier investigation reported the functional transfer of mitochondrial *rps1* to the nucleus in three genera of Trifolieae (*Trigonella*, *Melilotus* and *Medicago*) [39]. In the current study, the complete deletion of *rps1* gene from mitogenomes of four *Trifolium* species was detected (Figure S1), which is shared by the distantly related genus *Lotus*, a member of the tribe Loteae (Figure S2). There are two possible explanations for the phylogenetic distribution of the loss/transfer. The loss of mitochondrial *rps1* could be due to a single IGT in a common ancestor with differential resolution in descendant lineages, that is, acquisition of functional signals (or not) to stabilize transfer. Alternatively, there may have been independent functional transfers from an ancestor in each of the two unrelated lineages. To examine these alternatives, a maximum likelihood (ML) analysis was conducted using expanded taxon sampling of nuclear and mitochondrial *rps1* sequences. The resulting tree (Figure 5) included some long branches, which may be affected by the well-known phenomenon of long-branch attraction [52]. Nuclear *rps1* from *Lotus* and Trifolieae species were split into two independent clades, with intact and pseudogenized mitochondrial *rps1* placed between them. This pattern supports the explanation that functional transfers of *rps1* occurred at least two times in Papilionoideae, once in *Lotus* and a separate event in the ancestor of the Trifolieae clade that includes *Trigonella*, *Melilotus*, *Medicago* and *Trifolium*. The timing of the functional transfer of *rps1* in Trifolieae would likely be after the divergence of *Ononis* (Figure 5), which only has a mitochondrial copy [39].

Despite the putative functional replacement by nuclear *rps1*, the mitochondrial *rps1* in three genera (*Trigonella*, *Melilotus* and *Medicago*) was retained with limited sequence divergence (Figure 5), whereas it is completely and precisely deleted in *Trifolium* (Figure S1). Coding regions of plant mitogenomes are conserved by an accurate long homology-based repair mechanism, while non-coding regions are not conserved and are repaired by error-prone mechanisms [50]. Differential selection on mitogenomic molecules, which reduces harmful mutations on coding regions after double strand breaks (DSBs), was proposed to explain this [48,49]. Pseudogenized copies of mitochondrial *rps1* in the three genera *Trigonella*, *Melilotus* and *Medicago* are located adjacent to *nad5* exon1 (ca. 200 bp apart) [39]. Mutations in 5′ region of *nad5* exon1 that do not disturb transcription or translation of the functional gene and only affect pseudogenized *rps1* can be inherited by selection after DSBs. So, the adjacent location of mitochondrial *rps1* to *nad5* exon1 may enable retention of high sequence identity after functional replacement by sharing the benefit of accurate repair. A similar situation is known for the *rps14* pseudogene that is adjacent to *rpl5* in grasses [53]. Conservation of non-coding regions adjacent to coding regions is also present in mitogenome-wide sequence divergence comparisons across Fabaceae [8].

### 3.3. Shared DNA Among Genomes of Trifolium

Comparative analyses of the three genomic compartments (nuclear, mitochondrial and plastid) in *T. pratense* revealed a substantial amount of shared DNA between nuclear and organelle genomes, most of which was short fragments (Figure 4, Table 3). The shared DNAs between nuclear and mitochondrial genome was 135.4 kb (Figure 4) and had GC content more similar to those of mitogenomes (Table 1 and Table 3) suggesting that most IGT was unidirectional (i.e., mitochondrion to nucleus) and the nuclear genome of *T. pratense* includes numerous NUMTs. These NUMTs may integrate into the nuclear genome of *T. pratense* as short fragments. Alternatively, these short fragments may be the consequence of post-IGT mutational decay and rearrangement of longer NUMT sequences [54].

The discovery of a long stretch of NUMTs (spanning 348.5 kb; GC: 44.3%) in chromosome 4 of *T. repens* (Figure S3) supports a recent genomic scale IGT event. This type of large IGT was identified in *Arabidopsis thaliana* (Brassicaceae) in which ~270 kb of 367 kb mitogenome transferred to the nucleus [55] and covers an ~620 kb region of the nuclear genome [56]. To estimate the amount of NUMTs in *T. repens*, a mitogenome sequence from the same DNA source (white clover cv ‘Crau’ derivative) [46,57] is necessary. Large NUMTs were reported for animal nuclear genomes (little brown bat and fugu), however, these were later shown represent artifacts of genome assembly [58,59]. The nuclear genomes of *Trifolium* species are drafts with many gaps [43,44,45,46]. Verification of long putative NUMTs in *Trifolium* is needed to confirm genomic scale IGT events from the mitochondrial to nuclear genome.

### 3.4. Multiple Fissions of ccmF in Land Plants and a Novel Event in Trifolium

The first fission of mitochondrial *ccmF* dates back to the early evolution of land plants and split the gene into N-terminal (*ccmFn*) and C-terminal (*ccmFc*) coding regions [60]. In Marchantiales, the ORFs are closely adjacent (Figure S5). The mitogenome study of *Marchantia paleacea* (misidentified as *M. polymorpha* [61]) from the early 1990s [22] reported a fission of *ccmFc* (i.e., *ccmFc1* and *ccmFc2*) due to a single nucleotide deletion. This fission event was accepted in several subsequent papers [3,21,60], however, mitogenome sequences of two other *Marchantia* species (*M. inflexa* and *M. polymorpha* subsp. *ruderalis*) did not show the single nucleotide deletion, consistent with the other two available mitogenomes of Marchantiales (Figure S5). The initial report of a *ccmFc* fission in *Marchantia* should be re-examined to determine if it is specific to *M. paleacea* or the result of sequencing error.

In angiosperms, two independent fissions of *ccmFn* have been reported in *Allium* (Amaryllidaceae) [25] and Brassicaceae [24,62]. In both cases, *ccmFn1* and *ccmFn2* are distant from each other in the mitogenome and they share a similar breakpoint for the fission (Figure 6). The phylogenetic distribution of the fission in Amaryllidaceae was investigated by polymerase chain reaction using four genera in the family (*Narcissus*, *Tulbaghia*, *Ipheion* and *Allium*) and revealed that the separation of the two sequences is restricted to *Allium* [25]. However, the status of the other three genera without separation of *ccmFn* sequences does not necessarily guarantee that the gene is not split because there are cases of gene fission where the two new genes occupy a single locus, for example, fission of *ccmF* (into *ccmFn* and *ccmFc*) in Marchantiales (Figure S5) and *ccmFn* (into *ccmFn1* and *ccmFn2*) in *Trifolium* (Figure 2). The distribution and status of *ccmFn* fission in Amaryllidaceae needs further investigation including broad taxon sampling as well as confirmation with additional sequencing.

In Brassicaceae, it was argued that the fission is shared by all members of the family because it is present in five complete or draft mitochondrial genomes covering the earliest diverging genus (*Aethionema*) and other core genera (*Arabidopsis*, *Brassica*, *Raphanus*), whereas the mitogenome of the sister family Cleomaceae does not have the fission [62]. Further investigation, including additional published mitogenomes and assembled mitochondrial contigs for *ccmF* genes (Table S2), indicates that three species of *Aethionema* do not have the fission of *ccmFn* (Figure 6b). This discrepancy could be due an assembly error since the *Aethionema* data in the previous study was a draft mitogenome [62]. Whatever was the cause of discrepancy, it is clear that the fission of *ccmFn* is shared by many but not all Brassicaceae. The fission occurred after the divergence of *Aethionema* (Figure 6b); however, it is unknown if there was an intermediate stage that had experienced the fission but not physical separation of the *ccmFn1* and *ccmFn2*.

The independent fission of *ccmFn* in *Trifolium* represents a novel event. The fission was caused by a deletion of 59 bp resulting in a frame shift and premature stop codon (Figure 2). An alternative outcome of this deletion may be pseudogenization of the *ccmFn*. Mutational decay and deletion of pseudogenized mitochondrial genes can be delayed by proximity to functional genes (e.g., *rps1* in some Trifolieae genera and *rps14* in grasses, see Section 3.2). However, the gene that is consistently adjacent to *ccmFn* (*ccmFn1* and *ccmFn2*) is *ccmC*, which is ca. 8kb away from *ccmFn* in the four *Trifolium* species (Figure 1). Moreover, the expanded *ccmFn* sequence sampling confirms that the two ORFs (*ccmFn1* and *ccmFn2*) are conserved in eight *Trifolium* species with only a limited amount of sequence variation in coding regions (Figure S4). The fission break point in *Trifolium* is different from other angiosperms that express cytochrome c maturation protein from two ORFs, yet the conserved domains of the product remain intact (Figure 6c). Hence, the two ORFs of *ccmFn* are regarded as functional. The fission occurred after the divergence of genera *Trigonella* and *Melilotus* in the Trifolieae. The conserved adjacency of the two ORFs (*ccmFn1* and *ccmFn2*) may represent an early stage of the fission as in *ccmFn* and *ccmFc* in Marchantiales (Figure S5).

The fission of *ccmFn* in *Trifolium* leads to another question: is this event related to “intercompartmental piecewise gene transfer” [21]? To explore this question, we searched for ORFs of *ccmFn* in draft nuclear genomes of four *Trifolium* species (*T. subterraneum*, *T. pratense*, *T. pallescens* and *T. repens*). Both *T. pallescens* and *T. repens* (Figure S4) contained the *ccmFn* NUMTs however these were not restricted to a single ORF but included a locus covering both ORFs (*ccmFn1* and *ccmFn2*) and their flanking regions. The NUMTs were identical to their counterpart in mitogenome suggesting that the transfer was a recent event (or artifact in nuclear genome assembly, see discussion Section 3.3). Furthermore, there was no post-IGT sequence modification to suggest a functional transfer. Evidence did not support a relationship between fission of the mitochondrial gene *ccmFn* and piecewise or functional transfer in *Trifolium* species.

## 4. Materials and Methods

### 4.1. Assembly of Trifolium Mitogenomes

Four species of *Trifolium* from the two subgenera *Chronosemium* (*T. aureum* and *T. grandiflorum*) and *Trifolium* (*T. meduseum* and *T. pratense*) were selected for mitogenome assembly. The 100 bp paired-end raw Illumina (San Diego, CA, US) reads (Table 1) for mitogenome assembly were from Sabir et al. [31]. Assembly and mapping were conducted in Geneious Prime (https://www.geneious.com) using Geneious assembler and mapper, respectively. To assemble mitogenomes, the methods in Choi et al. [8] were followed. First, raw reads from the plastome were excluded by mapping total raw reads to corresponding plastomes [*T. aureum* (NC_024035.1), *T. grandiflorum* (NC_024034.1), *T. meduseum* (NC_024166.1) and *T. pratense* (MT039393)]. De novo assembly was subsequently conducted for each with ~30 million plastome-filtered reads. Among the assembled contigs, mitochondrial contigs were selected by BLAST searches against reference Fabaceae mitogenome sequences at National Center for Biotechnology Information (NCBI) (https://www.ncbi.nlm.nih.gov/genome/organelle/) using BLASTN 2.8.0+ [63] with default options. Mitochondrial contigs were manually assembled as single chromosomes in Geneious. Finally, draft mitogenomes were refined by mapping total plastome-filtered reads.

### 4.2. Annotation and Genome Content Comparison of Mitogenomes

To compare gene and intron content of *Trifolium* mitogenomes with related taxa, five previously published mitogenomes were acquired—two from IRLC [*Vicia faba* (KC189947) and *Medicago truncatula* (NC_029641)], one from the robinioid clade [*Lotus japonicus* (NC_016743)], which is sister to IRLC; and two from millettioid *sensu lato* clade [*Millettia pinnata* (NC_016742)] and *Glycine max* (NC_020455)], which is sister to the hologalegina clade (robinioid + IRLC). Annotation of rRNAs, protein coding genes and introns was conducted based on a reference mitogenome of *Liriodendron tulipifera* (NC_021152) with a set of 41 conserved mitochondrial genes in Geseq [64]. Annotation for protein coding genes was manually corrected in Geneious to fit ORFs. The annotation for tRNAs was cross-checked by tRNAscan-SE v2.0 [65].

### 4.3. Completion of the Trifolium Pratense Plastome

Plastome drafts of *Trifolium pratense* were reported in two different studies [31,34] but these sequences contained a complex repeat structure. Since these previous assemblies were based on short insert size data only (400-800 bp), the *T. pratense* plastome was redone using sequences generated from one of the previous studies [31] as well as mapping data from mate-pair Illumina sequences (ERX946087) with long insert sizes (7 kb) [43]. The newly assembled plastome was annotated as described above but with MPI-MP chloroplast references in GeSeq [64].

### 4.4. Repeat Estimation in Organelle Genomes

Repeat content was estimated in four mitogenomes and 13 plastomes (Table 2). Tandem repeats were identified using Tandem Repeats Finder version 4.09 [66] with default options. Other repeats (larger than 30 bp) were analyzed by BLASTN [63] searches using each genome as both subject and query with a word size of 7 and an e-value of 1e^−6^ as described in Guo et al. [67]. All BLAST hits were retained. Sequence coordinate information for BLAST hits was transferred to each genome as an annotation in Geneious and overlapping regions between hits were excluded from the estimations for repetitive DNA content. The distribution of dispersed repeat sequences across the genomes was visualized by Circoletto [68].

### 4.5. Shared DNA among Different Genomic Compartments

Shared DNA was evaluated in *Trifolium pratense* because this is the only species examined with completed sequences from all three genomic compartments. The mitogenome (MT039389) and plastome (MT039393) in this study were utilized and the nuclear genome was available as a chromosome-scale reference draft (LT990601- LT990607) [43]. Shared DNA among the genomes was evaluated in MegaBLAST with a word size of 28 and an e-value of 1e^−6^. For nuclear and organelle genome comparisons, each organelle sequence was used as the query against a subject database comprising the nuclear genome. For the comparison of organelle genomes, the plastome was used as the query and the mitogenome was the subject. BLAST hits with sequence identity higher than 90% were retained. Overlapping regions between hits were excluded from the estimations of shared DNA.

To search for putative large-scale IGT (> 100 kb) events, shared DNA analysis was conducted as described above but in this case the largest mitogenome (*T. meduseum*) and other published nuclear genomes of *Trifolium* (Table S1) were utilized. BLAST hits between the mitogenome and a long stretch of the nuclear region of *T. repens* were visualized by Circoletto [68].

### 4.6. Investigation on Status of rps1 in Nuclear and Mitochondrial Genome

Nuclear and mitochondrial sequences of *rps1* generated for a previous study [39] were acquired from NCBI. Nuclear *rps1* sequences for other species were searched by MegaBLAST using the options described above. Mitochondrial *rps1* of *Vicia faba* was used to query nuclear genomes of *Lotus japonicus*, *Medicago truncatula*, *Trifolium subterraneum*, *T. pratense*, *T. pallescens* and *T. repens* (Table S1). Mitochondrial *rps1* sequences were also extracted from mitogenomes of *Glycine max*, *Millettia pinnata*, *Vicia faba* and *Medicago truncatula*. All *rps1* sequences were aligned with MAFFT v.7.017 [69] using default options. Nucleotide substitution models were evaluated in jModelTest v.2.1.6 [70] by Akaike information criterion. ML analysis (GTR +G with 1000 bootstrap replications) was conducted using *G. max* and *M. pinnata* as outgroups in RAxML v.8 [71] in the CIPRES Science Gateway [72].

The status of mitochondrial *rps1* in *Trifolium* was tested by sequence alignment of the mitochondrial locus containing *rps1* and *nad5* exon1 in *M. truncatula* and the corresponding regions in four mitogenomes of *Trifolium*. Sequences were aligned in MAFFT [69] using default options followed by manual adjustments to minimize gaps and maximize apparent homologous regions.

### 4.7. Investigation of ccmF Fissions in Selected Land Plants

To investigate previously reported fission events of *ccmF* genes in land plants [22,23,24,25], all available sequences from published mitogenome sequences related to *Marchantia*, Brassicaceae, *Allium* (Amaryllidaceae) and Fabaceae were acquired. For *Marchantia*, published mitogenomes [22,73,74] of three species (*M. inflexa*, *M. polymorpha* subsp. *ruderalis* and *M. paleacea*) were examined: two from NCBI [*M. polymorpha* subsp. *ruderalis* (NC_037508.1) and *M. paleacea* (NC_001660.1)] and *M. inflexa*, which was downloaded from FigShare (https://figshare.com/articles/Marchantia_inflexa_mitochondrion_and_chloroplast_genomes/6639209/1). Two mitogenomes [*Dumortiera hirsuta* (NC_042873) and *Riccia fluitans* (NC_043906)], which are closely related to *Marchantia* in Marchantiales [75,76], were also included. For Amaryllidaceae, a single mitogenome [*Allium cepa* (NC_030100)] was available.

In addition to previously published and newly assembled mitogenomes, mitochondrial contigs were generated from available NGS reads for Brassicales and Fabaceae (Table S2). Raw sequences were mapped to reference *ccmF* sequences and the mapped reads were assembled in Geneious. The *ccmF* sequences of *Medicago truncatula* and *Batis maritima* were used as references for Fabaceae and Brassicales, respectively. Read depth of assembled *ccmF* genes (*ccmFn* and *ccmFc*) were compared to confirm that sequences originated from mitogenome rather than from other genomic compartments (i.e., nuclear and plastid genome). To search for nuclear copies of *ccmFn1* and *ccmFn2*, subject databases comprising four *Trifolium* nuclear genomes (Table S1) were queried with the mitochondrial *ccmFn* of *T. aureum* using MegaBLAST with default options. All sequences were aligned with MAFFT as described above. The status of *ccmFn* was plotted on cladograms from published phylogenetic studies of *Trifolium* [42] and Brassicaceae [47]. Conserved domains of *ccmFn* were detected using the Motif Scan of MyHits (http://myhits.isb-sib.ch/cgi-bin/motif_scan) [77,78].

## 5. Conclusions

The newly sequenced mitogenomes of *Trifolium* allowed comparative analyses of genome evolution for all three cellular compartments—mitochondrion, nucleus and plastid. Unlike many angiosperms, *Trifolium* lacks the highly repetitive genome organization of mitogenome. Some *Trifolium* plastomes has a much more complex organization and has accumulated more repeat contents than the mitogenome. A substantial amount of organellar DNA was detected in nuclear genomes of *Trifolium*, likely resulting from recent and nonfunctional IGT events. In addition, there has been an ancestral, functional transfer of mitochondrial *rps1* to the nuclear genome. A notable finding from the mitogenome of *Trifolium* was a novel gene fission of *ccmFn*. Analyses of *ccmF* genes in selected land plants provided further insights into the fission events. Although the current study is based on limited sampling of the three genomic compartments, our findings expand the understanding of how these genomes evolved in *Trifolium*. The underlying evolutionary and molecular mechanisms should be examined in future comparisons that incorporate broader taxonomic sampling for all three genomic compartments.

## Figures and Tables

**Table 1:** Assembly information for four *Trifolium* mitogenomes.

Species	Subgenus	Raw Reads	Mitogenome Coverage	Mitogenome Length (bp)	GC (%)	NCBI Accession
*T. aureum*	*Chronosemium*	71,346,514	645	294,911	44.9	MT039392
*T. grandiflorum*	*Chronosemium*	48,390,678	197	347,723	45.1	MT039391
*T. meduseum*	*Trifolium*	68,712,286	207	348,724	45.0	MT039390
*T. pratense*	*Trifolium*	47,909,108	212	301,823	45.2	MT039389

**Table 2:** Comparison of repeat percentage between organelle genomes in *Trifolium*.

Species	Subgenus	Section	Mitogenome			Plastome		
			Size (bp)	Repeat (%)	NCBI Accession	Size (bp)	Repeat (%)	NCBI Accession
*T. aureum*	*Chronosemium*	*Chronosemium*	294,911	8.6	MT039392	126,970	5.2	NC_024035
*T. grandiflorum*	*Chronosemium*	*Chronosemium*	347,723	8.4	MT039391	125,628	4.7	NC_024034
*T. boissieri*	*Chronosemium*	*Chronosemium*	Not applicable			125,740	5.1	NC_025743
*T. strictum*	*Trifolium*	*Paramesus*	Not applicable			125,834	4.4	NC_025745
*T. glanduliferum*	*Trifolium*	*Paramesus*	Not applicable			126,149	4.8	NC_025744
*T. lupinaster*	*Trifolium*	*Lupinaster*	Not applicable			135,049	10.9	KJ788287
*T. subterraneum*	*Trifolium*	*Trichocephalum*	Not applicable			144,763	19.7	NC_011828
*T. meduseum*	*Trifolium*	*Trichocephalum*	348,724	8.5	MT039390	142,595	19.5	NC_024166
*T. pratense*	*Trifolium*	*Trifolium*	301,823	6.6	MT039389	146,573	20.7	MT039393
*T. hybridum*	*Trifolium*	*Vesicastrum*	Not applicable			134,831	13.1	KJ788286
*T. semipilosum*	*Trifolium*	*Vesicastrum*	Not applicable			138,194	15.8	KJ788291
*T. repens*	*Trifolium*	*Trifoliastrum*	Not applicable			132,120	10.7	NC_024036
*T. occidentale*	*Trifolium*	*Trifoliastrum*	Not applicable			133,806	11.1	KJ788289

**Table 3:** BLAST hit statistics for shared DNA between nuclear and organelle genomes in *Trifolium pratense*.

Comparison	Number	Average Identity	GC (%)	Length (bp)			
				Min	Max	Mean	Median
Nuclear vs. Mitochondria	1830	95.5	45.8	33	3950	121.8	93.5
Nuclear vs. Plastid	1086	95.7	35.1	34	2027	144.2	118.5

## Supplementary Materials

Supplementary Materials can be found at https://www.mdpi.com/1422-0067/21/6/1959/s1. Figure S1. Nucleotide alignment showing deletion of mitochondrial *rps1* in *Trifolium* species. Figure S2. Gene and cis-spliced intron content across six Papilionoideae genera. Figure S3. Circoletto map showing similar sequences between mitogenome of *T. meduseum* (left arc) and a continuous region of nuclear genome (right arc) of *T. repens* (chromosome 4; NCBI accession: VCDJ01010667; position: 72,476,623-72,825,180). Figure S4. Sequence variation of *ccmFn* in *Trifolium* species. Figure S5. Alignment of the mitochondrial region containing *ccmFn* and *ccmFc* genes from five species of Marchantiales. Table S1. Information on nuclear genomes, used for comparative study. Table S2. List of taxa for *ccmF* analysis with information about sequence sources and status of the genes.

## Abbreviations

IGT	Intracellular gene transfer
NUMT	Nuclear mitochondrial DNA sequences
NUPT	Nuclear plastid DNA sequences
IR	Inverted repeat
IRLC	Inverted repeat lacking clade
ORF	Open reading frame
NGS	Next-generation sequencing
DNA-RRR	DNA replication, recombination and repair
ML	Maximum likelihood
DSB	Double strand break
NCBI	National Center for Biotechnology Information
