# Supplementary material for: Comparative Mitogenome Analysis of the Genus Trifolium Reveals Independent Gene Fission of ccmFn and Intracellular Gene Transfers in Fabaceae
Source: Int J Mol Sci. 2020 Mar 13;21(6):1959. doi: 10.3390/ijms21061959 (PMC7139807; doi:10.3390/ijms21061959)
Supplement: Supplementary file 1 [file ijms-21-01959-s001.zip › Supplementary_Figures_re.docx]

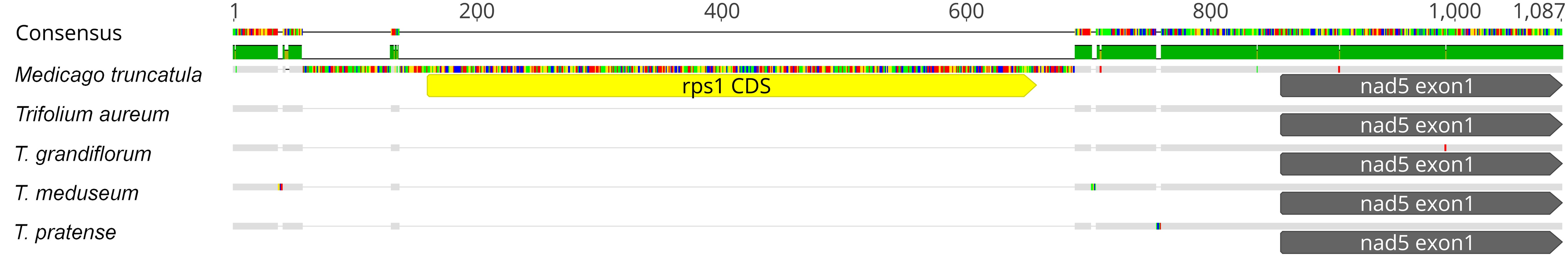


**Figure S1.** Nucleotide alignment showing deletion of mitochondrial *rps1* in *Trifolium* species. Translated amino acid sequence is presented below the mitochondrial *rps1* of *Medicago truncatula*. Nucleotide coordinates are indicated on consensus alignment. Sequence identity is shown below consensus (green = 100%, yellow-green = at least 30% and under 100%, red = below 30%).


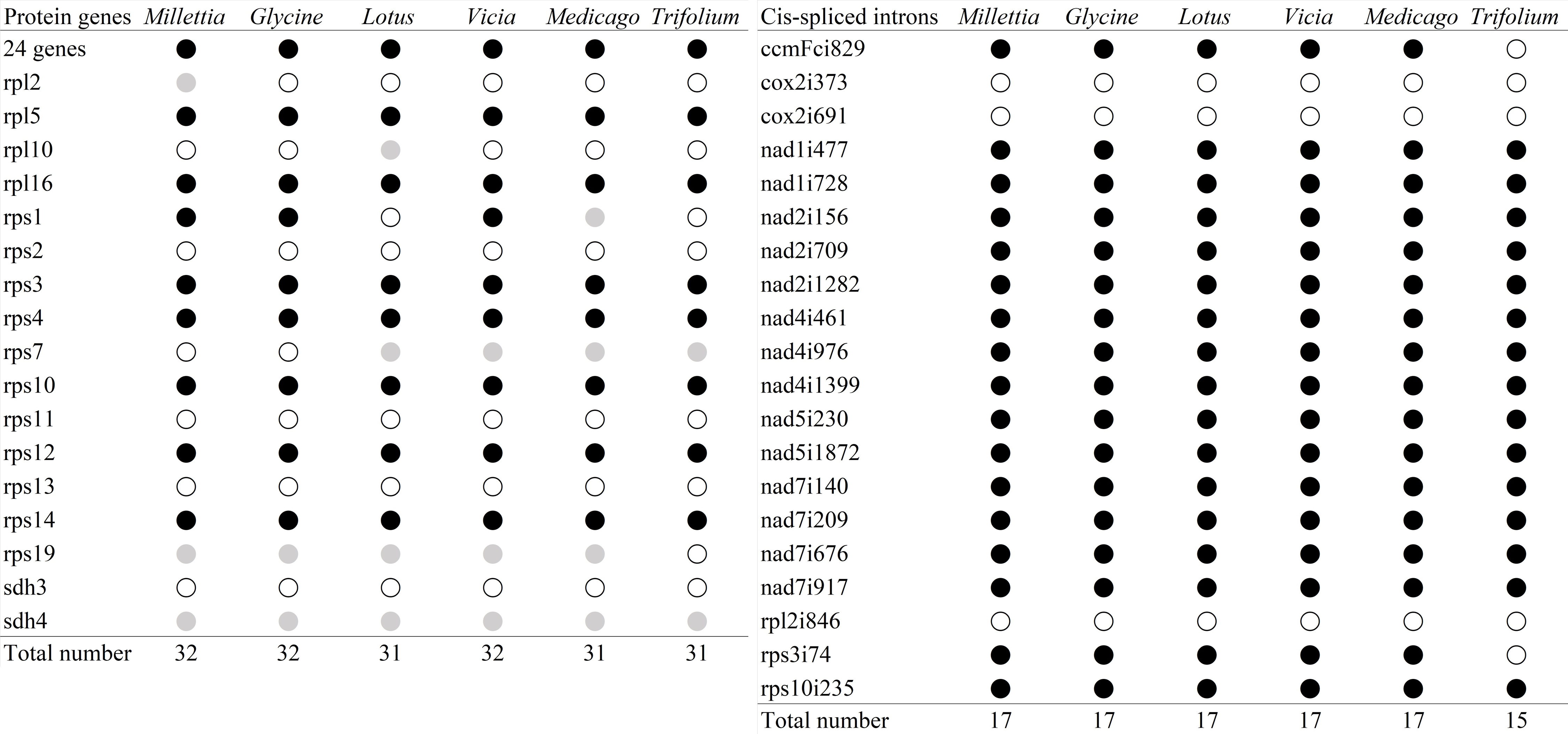


**Figure S2.** Gene and cis-spliced intron content across six Papilionoideae genera. The 24 conserved genes are *atp* (*1*, *4*, *6*, *8*, *9*), *ccm* (*B*, *C*, *Fn*, *Fc*), *cob*, *cox* (*1*, *2*, *3*), *matR*, *mttB*, *nad* (*1*, *2*, *3*, *4*, *4L*, *5*, *6*, *7*, *9*). The two ORFs of *ccmFn* (*ccmFn1* and *ccmFn2*) are treated as single gene here. For gene content, black closed circle = intact, gray closed circle = pseudogene, open circle = absent. For intron content, closed circle = present, open circle = absent.


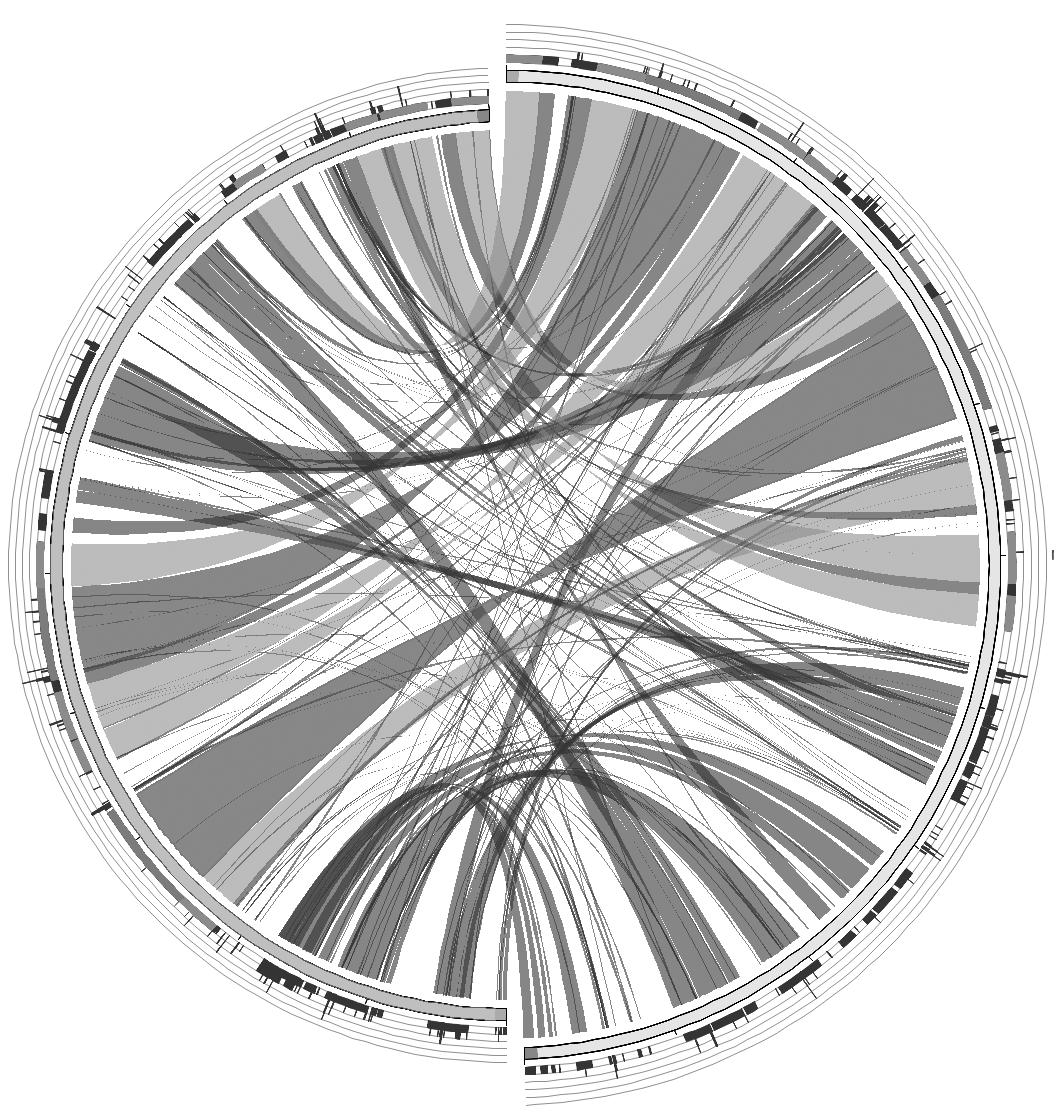


**Figure S3**. Circoletto map showing similar sequences between the mitogenome of *T. meduseum* (left arc) and a continuous region of nuclear genome (right arc) of *T. repens* (chromosome 4; NCBI accesion: VCDJ01010667; position: 72,476,623-72,825,180). Each ribbon represents a BLAST hit between two sequences. Multiple hits in a single region are indicated by histograms in outer concentric rings.


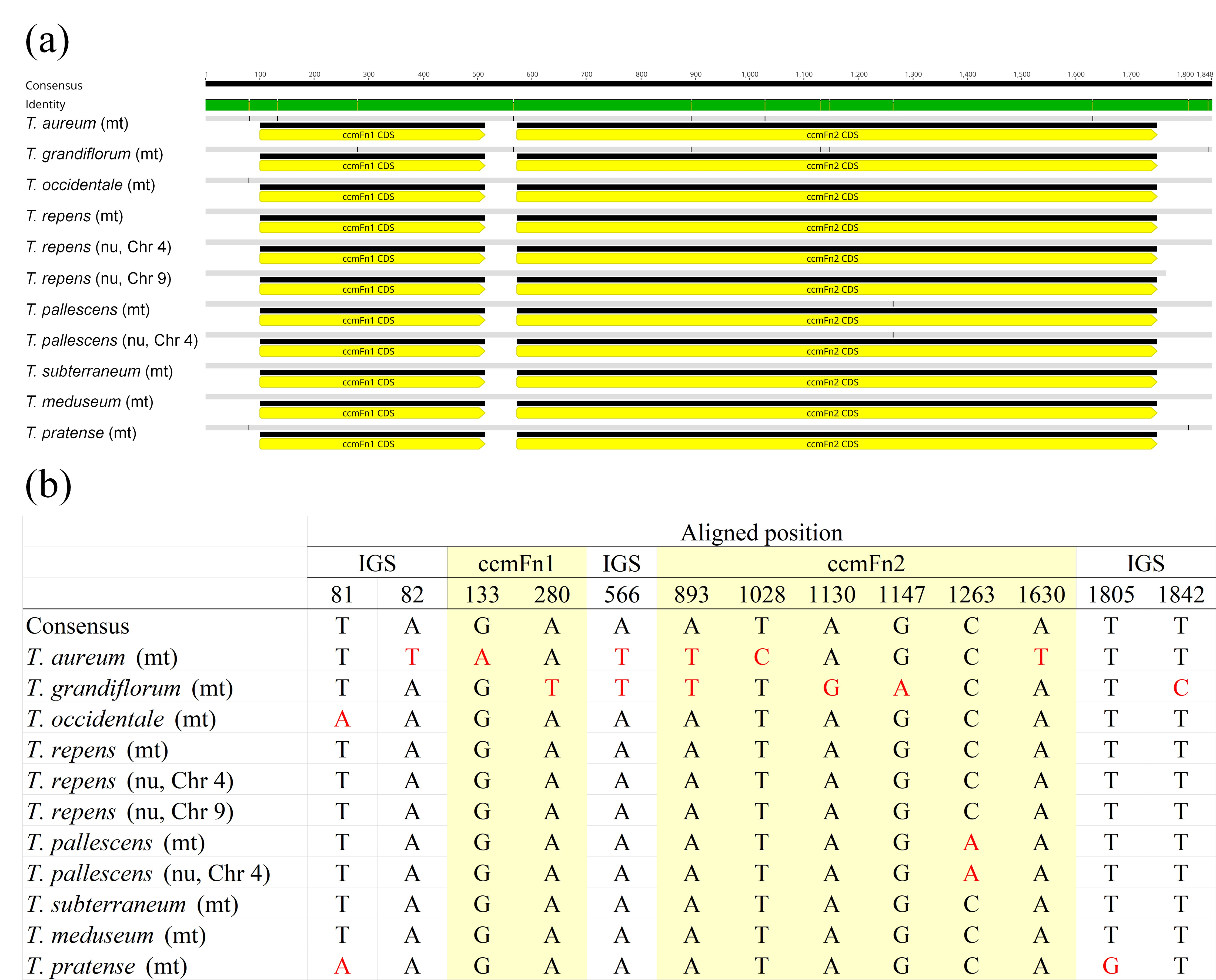


**Figure S4.** Sequence variation of *ccmFn* in *Trifolium* species. Sources of the sequences are indicated in the parentheses (mt = mitochondria and nu = nucleus). The chromosome numbers are indicated for nuclear copies. (a) Full-length alignment of 11 sequences of *ccmFn* (*ccmFn1* and ccmFn2) and its 100 bp flanking regions. Translated amino acid sequences are presented below corresponding aligned DNA sequences. Nucleotide coordinates are indicated above consensus alignment. Sequence identity is shown below consensus (green = 100%, yellow-green = at least 30% and under 100%, red = below 30%). (b) Summary of variation in sequence alignment. Sequences different from consensus are marked with red font. Aligned region for protein coding region is in yellow.


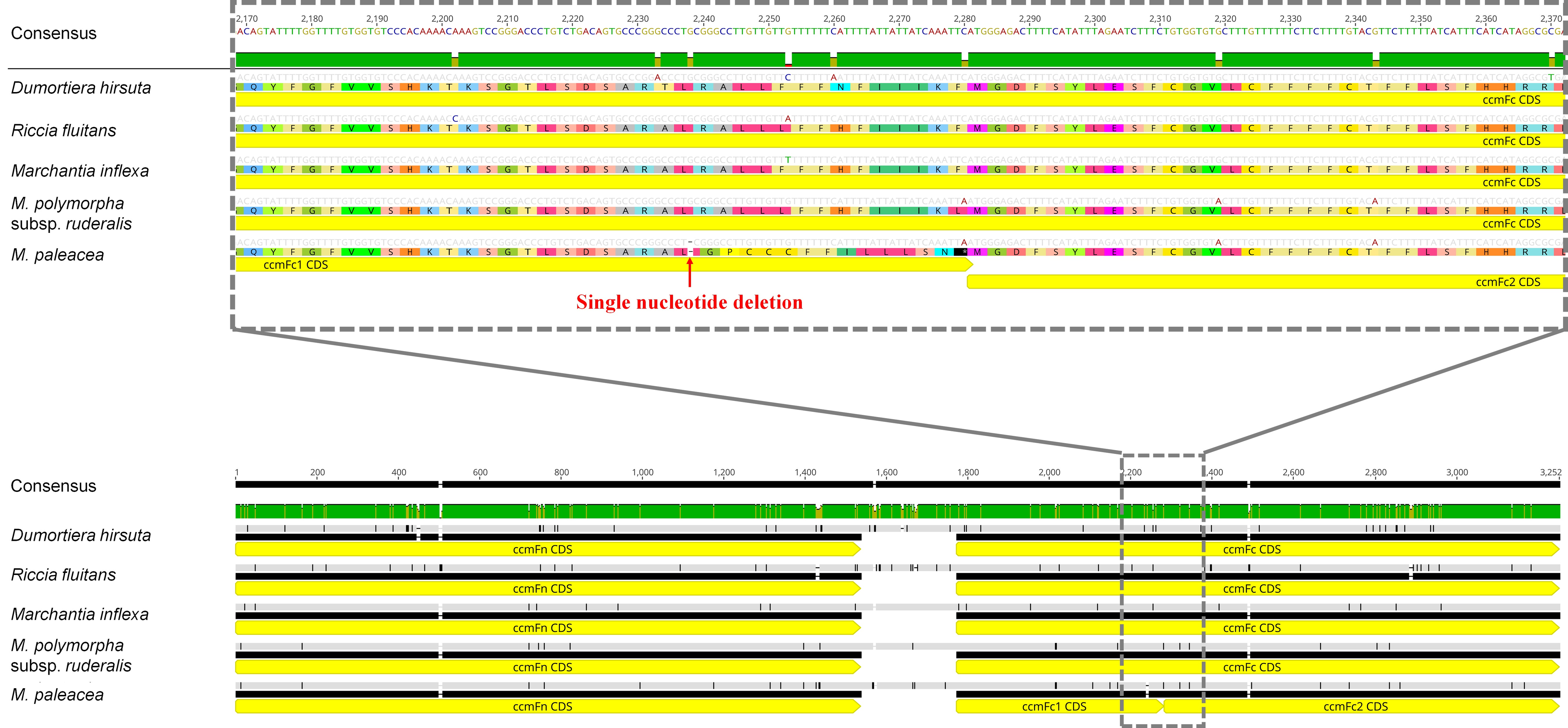


**Figure S5.** Alignment of the mitochondrial region containing *ccmFn* and *ccmFc* genes from five species of Marchantiales. The region (dashed gray rectangle; aligned position 2169-2372) including the single nucleotide deletion is on the upper part. Translated amino acid sequences are presented below each of corresponding aligned DNA sequences. Nucleotide coordinates are indicated above consensus alignment. Sequence identity is shown below consensus (green = 100%, yellow-green = at least 30% and under 100%, red = below 30%).
